# 1-(4-Fluoro­phen­yl)-3-hydr­oxy-3-phenyl­prop-2-en-1-one

**DOI:** 10.1107/S1600536809004747

**Published:** 2009-02-13

**Authors:** Chun-Yang Zheng, Dun-Jia Wang, Ling Fan

**Affiliations:** a Hubei Key Laboratory of Pollutant Analysis & Reuse Technology, Hubei Normal University, Huangshi 435002,People’s Republic of China; b College of Chemistry and Environmental Engineering, Hubei Normal University, Huangshi 435002, People’s Republic of China

## Abstract

In the crystal structure the title compound, C_15_H_11_FO_2_, the molecule exists in the enol form. It is stabilized by an intra­molecular O—H⋯O hydrogen bond, in which the donor O—H and acceptor H⋯O distances are almost equal. The dihedral angle between the two benzene rings is 22.30 (4)°.

## Related literature

For background to the uses and characteristics of 1,3-diketones, see: Gilli *et al.* (2004 ▶); Hasegawa *et al.* (1997 ▶); Jang *et al.* (2006 ▶); Ma *et al.* (1999 ▶); Yoshida *et al.* (2005 ▶). For geometric data, see: Bertolasi *et al.* (1991 ▶); Wang *et al.* (2006 ▶).
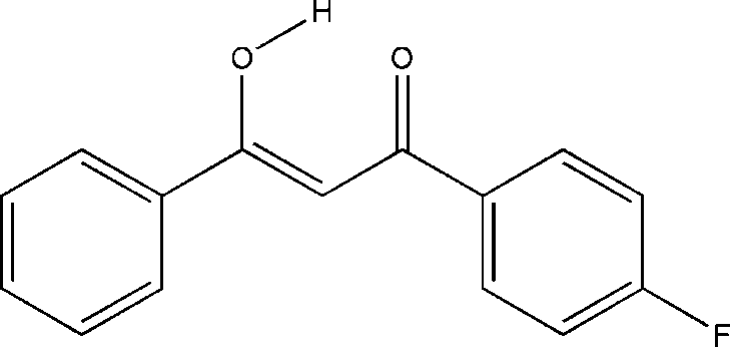


## Experimental

### 

#### Crystal data

C_15_H_11_FO_2_
*M* *_r_* = 242.24
Monoclinic, 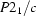

*a* = 11.8526 (5) Å
*b* = 11.7192 (5) Å
*c* = 9.4164 (4) Å
β = 113.405 (1)°
*V* = 1200.35 (9) Å^3^
*Z* = 4
Mo *K*α radiation
μ = 0.10 mm^−1^
*T* = 298 K
0.30 × 0.10 × 0.04 mm

#### Data collection

Bruker SMART CCD area-detector diffractometer
Absorption correction: multi-scan (*SADABS*; Sheldrick, 1996 ▶) *T*_min_ = 0.991, *T*_max_ = 0.996
11206 measured reflections
2109 independent reflections
1295 reflections with *I* > 2σ(*I*)
*R*_int_ = 0.106

#### Refinement

*R*[*F* ^2^ > 2σ(*F* ^2^)] = 0.051
*wR*(*F* ^2^) = 0.140
*S* = 0.93
2109 reflections
166 parameters
H atoms treated by a mixture of independent and constrained refinement
Δρ_max_ = 0.25 e Å^−3^
Δρ_min_ = −0.19 e Å^−3^

### 

Data collection: *SMART* (Bruker, 1997 ▶); cell refinement: *SAINT* (Bruker, 1999 ▶); data reduction: *SAINT*; program(s) used to solve structure: *SHELXS97* (Sheldrick, 2008 ▶); program(s) used to refine structure: *SHELXL97* (Sheldrick, 2008 ▶); molecular graphics: *SHELXTL* (Sheldrick, 2008 ▶); software used to prepare material for publication: *SHELXTL*.

## Supplementary Material

Crystal structure: contains datablocks global, I. DOI: 10.1107/S1600536809004747/kj2112sup1.cif

Structure factors: contains datablocks I. DOI: 10.1107/S1600536809004747/kj2112Isup2.hkl

Additional supplementary materials:  crystallographic information; 3D view; checkCIF report

## Figures and Tables

**Table 1:** Hydrogen-bond geometry (Å, °)

*D*—H⋯*A*	*D*—H	H⋯*A*	*D*⋯*A*	*D*—H⋯*A*
O2—H2*A*⋯O1	1.23 (3)	1.30 (3)	2.4827 (19)	157 (2)

**Fig. 1.**
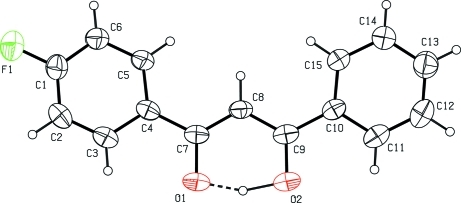
View of the title compound, showing the atom-labeling scheme. Displacement ellipsoids are drawn at the 50% probability level.

## supplementary crystallographic information

### Comment

1,3-Diketones posses a broad spectrum of useful and sometimes unique chemical properties, which make them extremely attractive as intermediates in syntheses (Hasegawa *et al.*,  1997). They are also used in the chemistry of metallocomplexes (Ma *et al.*,  1999; Yoshida *et al.*, 2005; Jang *et al.*,  2006). 1,3-Diketone structures have received increasing attention due to their enolic tautomeric forms and their ability to form strong intermolecular or intramolecular hydrogen bonds (Gilli *et al.*,  2004). The crystal structure of the title compound (Fig. 1) is in the enol form, stabilized by an intramolecular hydrogen bond (Table 2). The bond lengths in the diketone fragment are either significantly shorter than normal single bonds or significantly longer than normal double bonds (Table 1). This shows that the structure displays a strong delocalization of double bonds in this region. The geometric data are in agreement with reported literature values (Bertolasi *et al.*,  1991; Wang *et al.*,  2006). The dihedral angle between the two aromatic rings is 22.30 (4)°.

### Experimental

1-(4-Fluorophenyl)ethanone (1.38 g, 0.01 mol), ethyl benzoate (1.50 g, 0.01 mol), NaNH_2_ (0.78 g, 0.02 mol) and dry ether (40 ml) were placed in a round bottom flask. The mixture was stirred 6 h at room temperature under a blanket of nitrogen, acidified with dilute hydrochloric acid, and stirring was continued until all solids dissolved. The ether layer was separated and washed with saturated NaHCO_3_ solution, dried over anhydrous Na_2_SO_4_ and the solvent was removed by evaporation. The residual solid was recrystallized from an ethanol solution to give the title compound (yield 1.27 g, 52.4%, m.p. 351 K). Crystals suitable for X-ray diffraction were grown by slow evaporation of CHCl_2_–EtOH (1:5) solutions at room temperature.

### Refinement

The H atom of the hydroxyl group was located in a difference Fourier map and its position was refined freely, with *U*_iso_(H) = 1.5 *U*_iso_(O). The other H atoms were placed in geometrically idealized positions and constrained to ride on their parent atoms, with C—H distances of 0.93 to 0.97 Å, and with *U*_iso_(H) = 1.2 *U*_eq_(C).

### Crystal data

**Table:**

C_15_H_11_FO_2_	*F*(000) = 504
*M**_r_* = 242.24	*D*_x_ = 1.340 Mg m^−^^3^
Monoclinic, *P*2_1_/*c*	Melting point: 351 K
Hall symbol: -P2ybc	Mo *K*α radiation, λ = 0.71073 Å
*a* = 11.8526 (5) Å	Cell parameters from 2616 reflections
*b* = 11.7192 (5) Å	θ = 2.6–22.7°
*c* = 9.4164 (4) Å	µ = 0.10 mm^−^^1^
β = 113.405 (1)°	*T* = 298 K
*V* = 1200.35 (9) Å^3^	Plate, colourless
*Z* = 4	0.30 × 0.10 × 0.04 mm

### Data collection

**Table:**

Bruker SMART CCD area-detector diffractometer	2109 independent reflections
Radiation source: fine-focus sealed tube	1295 reflections with *I* > 2σ(*I*)
graphite	*R*_int_ = 0.106
φ and ω scans	θ_max_ = 25.0°, θ_min_ = 1.9°
Absorption correction: multi-scan (*SADABS*; Sheldrick, 1996)	*h* = −14→14
*T*_min_ = 0.991, *T*_max_ = 0.996	*k* = −13→13
11206 measured reflections	*l* = −11→11

### Refinement

**Table:**

Refinement on *F*^2^	Primary atom site location: structure-invariant direct methods
Least-squares matrix: full	Secondary atom site location: difference Fourier map
*R*[*F*^2^ > 2σ(*F*^2^)] = 0.051	Hydrogen site location: inferred from neighbouring sites
*wR*(*F*^2^) = 0.140	H atoms treated by a mixture of independent and constrained refinement
*S* = 0.93	*w* = 1/[σ^2^(*F*_o_^2^) + (0.0849*P*)^2^] where *P* = (*F*_o_^2^ + 2*F*_c_^2^)/3
2109 reflections	(Δ/σ)_max_ < 0.001
166 parameters	Δρ_max_ = 0.25 e Å^−^^3^
0 restraints	Δρ_min_ = −0.19 e Å^−^^3^

### Special details

**Table:**

Geometry. All e.s.d.'s (except the e.s.d. in the dihedral angle between two l.s. planes) are estimated using the full covariance matrix. The cell e.s.d.'s are taken into account individually in the estimation of e.s.d.'s in distances, angles and torsion angles; correlations between e.s.d.'s in cell parameters are only used when they are defined by crystal symmetry. An approximate (isotropic) treatment of cell e.s.d.'s is used for estimating e.s.d.'s involving l.s. planes.
Refinement. Refinement of *F*^2^ against ALL reflections. The weighted *R*-factor *wR* and goodness of fit *S* are based on *F*^2^, conventional *R*-factors *R* are based on *F*, with *F* set to zero for negative *F*^2^. The threshold expression of *F*^2^ > σ(*F*^2^) is used only for calculating *R*-factors(gt) *etc*. and is not relevant to the choice of reflections for refinement. *R*-factors based on *F*^2^ are statistically about twice as large as those based on *F*, and *R*- factors based on ALL data will be even larger.

### Fractional atomic coordinates and isotropic or equivalent isotropic displacement parameters (Å^2^)

**Table:**

	*x*	*y*	*z*	*U*_iso_*/*U*_eq_	
C1	0.27135 (19)	0.29172 (18)	0.4883 (2)	0.0742 (6)	
C2	0.29882 (19)	0.17926 (17)	0.4841 (2)	0.0804 (6)	
H2	0.2565	0.1236	0.5136	0.096*	
C3	0.38951 (19)	0.14902 (16)	0.4358 (2)	0.0753 (6)	
H3	0.4082	0.0722	0.4329	0.090*	
C4	0.45487 (16)	0.23125 (14)	0.39078 (19)	0.0600 (5)	
C5	0.42305 (18)	0.34513 (15)	0.3965 (2)	0.0694 (6)	
H5	0.4645	0.4018	0.3673	0.083*	
C6	0.33131 (19)	0.37574 (17)	0.4446 (2)	0.0781 (6)	
H6	0.3106	0.4520	0.4473	0.094*	
C7	0.55320 (17)	0.19568 (14)	0.3396 (2)	0.0634 (5)	
C8	0.63154 (16)	0.27122 (14)	0.3102 (2)	0.0627 (5)	
H8	0.6220	0.3491	0.3208	0.075*	
C9	0.72513 (17)	0.23318 (15)	0.2648 (2)	0.0664 (5)	
C10	0.81581 (16)	0.30949 (15)	0.2408 (2)	0.0635 (5)	
C11	0.8874 (2)	0.26895 (18)	0.1647 (2)	0.0811 (6)	
H11	0.8778	0.1941	0.1291	0.097*	
C12	0.9732 (2)	0.3393 (2)	0.1414 (3)	0.0959 (7)	
H12	1.0190	0.3116	0.0883	0.115*	
C13	0.99055 (19)	0.4487 (2)	0.1958 (3)	0.0909 (7)	
H13	1.0486	0.4952	0.1811	0.109*	
C14	0.92093 (19)	0.48972 (18)	0.2731 (2)	0.0901 (7)	
H14	0.9329	0.5639	0.3112	0.108*	
C15	0.83398 (19)	0.42134 (17)	0.2941 (2)	0.0781 (6)	
H15	0.7869	0.4505	0.3445	0.094*	
F1	0.18286 (12)	0.32154 (11)	0.53824 (16)	0.1081 (5)	
O1	0.56461 (14)	0.08669 (11)	0.32400 (18)	0.0923 (5)	
O2	0.73642 (15)	0.12538 (11)	0.24314 (19)	0.0931 (5)	
H2A	0.650 (2)	0.087 (2)	0.270 (3)	0.140*	

### Atomic displacement parameters (Å^2^)

**Table:**

	*U*^11^	*U*^22^	*U*^33^	*U*^12^	*U*^13^	*U*^23^
C1	0.0767 (13)	0.0819 (14)	0.0718 (13)	−0.0013 (11)	0.0378 (11)	0.0039 (10)
C2	0.0866 (15)	0.0725 (14)	0.0856 (15)	−0.0167 (11)	0.0379 (12)	0.0098 (11)
C3	0.0870 (14)	0.0523 (12)	0.0853 (14)	−0.0058 (10)	0.0329 (12)	0.0056 (9)
C4	0.0695 (11)	0.0483 (10)	0.0548 (11)	−0.0051 (8)	0.0170 (9)	0.0040 (8)
C5	0.0849 (14)	0.0483 (11)	0.0837 (14)	−0.0008 (9)	0.0428 (11)	0.0040 (9)
C6	0.0959 (15)	0.0591 (12)	0.0938 (15)	0.0021 (10)	0.0530 (13)	0.0009 (10)
C7	0.0750 (12)	0.0442 (10)	0.0667 (12)	0.0046 (9)	0.0235 (10)	0.0011 (8)
C8	0.0714 (12)	0.0430 (10)	0.0733 (12)	0.0053 (8)	0.0284 (10)	−0.0035 (8)
C9	0.0777 (13)	0.0523 (11)	0.0640 (12)	0.0101 (9)	0.0227 (10)	−0.0037 (9)
C10	0.0660 (12)	0.0605 (12)	0.0621 (11)	0.0125 (9)	0.0236 (9)	−0.0006 (9)
C11	0.0863 (14)	0.0781 (14)	0.0830 (15)	0.0148 (12)	0.0380 (12)	−0.0110 (11)
C12	0.0910 (16)	0.114 (2)	0.0986 (18)	0.0141 (14)	0.0549 (15)	−0.0037 (14)
C13	0.0850 (15)	0.0948 (17)	0.0980 (17)	0.0036 (13)	0.0418 (14)	0.0061 (13)
C14	0.0968 (15)	0.0715 (13)	0.1198 (19)	−0.0030 (11)	0.0617 (15)	−0.0042 (12)
C15	0.0875 (14)	0.0619 (12)	0.1025 (16)	0.0039 (10)	0.0562 (13)	−0.0060 (11)
F1	0.1104 (10)	0.1162 (11)	0.1240 (11)	−0.0001 (8)	0.0742 (9)	0.0031 (8)
O1	0.1175 (12)	0.0406 (8)	0.1328 (13)	0.0027 (7)	0.0647 (10)	−0.0009 (7)
O2	0.1094 (12)	0.0522 (8)	0.1332 (13)	0.0088 (7)	0.0645 (11)	−0.0136 (8)

### Geometric parameters (Å, °)

**Table:**

C1—F1	1.354 (2)	C8—H8	0.9300
C1—C2	1.362 (3)	C9—C10	1.483 (3)
C1—C6	1.370 (3)	C10—C15	1.389 (3)
C2—C3	1.369 (3)	C10—C11	1.393 (3)
C2—H2	0.9300	C11—C12	1.393 (3)
C3—C4	1.404 (3)	C11—H11	0.9300
C3—H3	0.9300	C12—C13	1.365 (3)
C4—C5	1.394 (2)	C12—H12	0.9300
C4—C7	1.487 (3)	C13—C14	1.386 (3)
C5—C6	1.381 (3)	C13—H13	0.9300
C5—H5	0.9300	C14—C15	1.380 (3)
C6—H6	0.9300	C14—H14	0.9300
O1—C7	1.299 (2)	C15—H15	0.9300
O2—C9	1.295 (2)	O1—H2A	1.30 (3)
C7—C8	1.388 (2)	O2—H2A	1.23 (3)
C8—C9	1.410 (3)		
			
F1—C1—C2	119.14 (18)	O2—C9—C8	119.94 (18)
F1—C1—C6	118.98 (18)	O2—C9—C10	115.95 (17)
C2—C1—C6	121.88 (18)	C8—C9—C10	124.11 (16)
C1—C2—C3	119.24 (18)	C15—C10—C11	117.95 (18)
C1—C2—H2	120.4	C15—C10—C9	122.10 (16)
C3—C2—H2	120.4	C11—C10—C9	119.94 (17)
C2—C3—C4	121.53 (19)	C12—C11—C10	120.73 (19)
C2—C3—H3	119.2	C12—C11—H11	119.6
C4—C3—H3	119.2	C10—C11—H11	119.6
C5—C4—C3	117.09 (18)	C13—C12—C11	120.5 (2)
C5—C4—C7	122.68 (16)	C13—C12—H12	119.8
C3—C4—C7	120.22 (17)	C11—C12—H12	119.8
C6—C5—C4	121.45 (18)	C12—C13—C14	119.4 (2)
C6—C5—H5	119.3	C12—C13—H13	120.3
C4—C5—H5	119.3	C14—C13—H13	120.3
C1—C6—C5	118.82 (18)	C15—C14—C13	120.5 (2)
C1—C6—H6	120.6	C15—C14—H14	119.7
C5—C6—H6	120.6	C13—C14—H14	119.7
O1—C7—C8	119.79 (17)	C14—C15—C10	120.90 (18)
O1—C7—C4	116.27 (16)	C14—C15—H15	119.5
C8—C7—C4	123.94 (15)	C10—C15—H15	119.5
C7—C8—C9	121.86 (16)	C7—O1—H2A	99.8 (11)
C7—C8—H8	119.1	C9—O2—H2A	100.1 (12)
C9—C8—H8	119.1		
			
F1—C1—C2—C3	−179.05 (18)	C4—C7—C8—C9	178.70 (16)
C6—C1—C2—C3	0.5 (3)	C7—C8—C9—O2	2.9 (3)
C1—C2—C3—C4	0.0 (3)	C7—C8—C9—C10	−176.33 (17)
C2—C3—C4—C5	−0.3 (3)	O2—C9—C10—C15	−164.43 (17)
C2—C3—C4—C7	179.74 (17)	C8—C9—C10—C15	14.9 (3)
C3—C4—C5—C6	0.1 (3)	O2—C9—C10—C11	14.5 (3)
C7—C4—C5—C6	−179.97 (17)	C8—C9—C10—C11	−166.16 (17)
F1—C1—C6—C5	178.82 (17)	C15—C10—C11—C12	−0.8 (3)
C2—C1—C6—C5	−0.7 (3)	C9—C10—C11—C12	−179.86 (19)
C4—C5—C6—C1	0.4 (3)	C10—C11—C12—C13	1.5 (3)
C5—C4—C7—O1	−172.91 (17)	C11—C12—C13—C14	−0.8 (4)
C3—C4—C7—O1	7.0 (2)	C12—C13—C14—C15	−0.6 (4)
C5—C4—C7—C8	7.8 (3)	C13—C14—C15—C10	1.2 (3)
C3—C4—C7—C8	−172.31 (18)	C11—C10—C15—C14	−0.5 (3)
O1—C7—C8—C9	−0.6 (3)	C9—C10—C15—C14	178.53 (19)

### Hydrogen-bond geometry (Å, °)

**Table:**

*D*—H···*A*	*D*—H	H···*A*	*D*···*A*	*D*—H···*A*
O2—H2A···O1	1.23 (3)	1.30 (3)	2.4827 (19)	157 (2)
